# A randomised controlled trial in preterm infants comparing prophylactic with selective “less invasive surfactant administration” (pro.LISA)

**DOI:** 10.1186/s13063-023-07603-7

**Published:** 2023-09-26

**Authors:** Wolfgang Göpel, Tanja K. Rausch, Barbara Mitschdörfer, Silke Mader, Egbert Herting, Inke R. König, Guido Stichtenoth, on behalf of the pro.LISA study group, Thomas Höhn, Anja Stein, Hagen Bayer, Susanne Schmidtke, Barabara Naust, Claudia Roll, Axel Franz, Moritz Wolff, Anna Siemes, Katja Schneider, Jana Katharina Dieks, Hans Fuchs, Thorsten Körner, Michael Schroth, Frank Dohle, Thomas Völkl, Christoph Härtel, Levente Bejo, Welfhard Schneider

**Affiliations:** 1 Department of Pediatrics, University Hospital Schleswig-Holstein, University of Lübeck, Lübeck, Germany; 2 Institute of Medical Biometry and Statistics, University of Lübeck, Lübeck, Germany; 3 Bundesverband “Das Frühgeborene Kind”, Frankfurt, Germany; 4 European Foundation for the Care of Newborn Infants (EFCNI), Munich, Germany

**Keywords:** Surfactant, Preterm infant, LISA

## Abstract

**Background:**

Respiratory distress syndrome is the main cause of mortality and morbidity in preterm infants. “Less invasive surfactant administration” (LISA), which describes intratracheal surfactant administration to spontaneously breathing infants via a small diameter tube, is recommended as the first-line treatment in preterm infants with more than 30% supplemental oxygen. Prophylactic use of LISA in preterm infants with less than 30% supplemental oxygen was not tested in randomised controlled trials yet, and long-term outcome data of the procedure are scarce.

**Methods:**

Preterm infants with a gestational age between 25 weeks + 0 days and 28 weeks + 6 days who are breathing spontaneously on continuous positive airway pressure with supplemental oxygen at or below 30% in the first hour of life will be randomised to a prophylactic LISA treatment with 100–200 mg surfactant intratracheally per kilogramme bodyweight (intervention group) or will continue the continuous positive airway pressure treatment (control group). Participants will have follow-up until age 5 years. At that time, the children will be tested by spirometry, and forced expiratory volume within 1-s *z*-scores will be compared between the intervention and control groups as the primary outcome parameter of the trial. Secondary endpoints include additional lung function parameters, endurance, motor development, intelligence, and sensitivity for infectious lung diseases. Short-term safety assessment will be done after completed enrolment (*n* = 698) and discharge of all infants. This safety assessment will include in-hospital mortality and short-term complications.

**Discussion:**

Robust data concerning the possible long-term benefits of prophylactic LISA treatment are lacking. The current observational data from the German Neonatal Network indicate that approximately 50% of preterm infants with supplemental oxygen at or below 30% within the first hour of life are treated with LISA. The pro.LISA trial will provide short- and long-term outcomes of preterm infants receiving prophylactic treatment and will clarify if prophylactic treatment should be given to all preterm infants or if the current practice of selective treatment if supplemental oxygen exceeds 30% is more appropriate.

**Trial registration:**

German Clinical Trials Register DRKS00028086. Prospectively registered on 8 February 2022.

## Administrative information

Note: The numbers in curly brackets in this protocol refer to the SPIRIT checklist item numbers. The order of the items has been modified to group similar items (see http://www.equator-network.org/reporting-guidelines/spirit-2013-statement-defining-standard-protocol-items-for-clinical-trials/).

**Table:** 

Title {1}	A randomised controlled trial in preterm infants comparing prophylactic with selective “less invasive surfactant administration” (pro.LISA)
Trial registration {2a and 2b}	German Clinical Trials Register as study number DRKS00028086. The German Clinical Trial Register collects all items from the WHO Trial Registration Data Set.
Protocol version {3}	Pro.LISA_01_21 version 4, from 11th January 2023
Funding {4}	Federal Ministry of Education and Research (BMBF) Funding no.: 01KG2024
Author details {5}	Wolfgang Göpel, Egbert Herting and Guido Stichtenoth: Department of Pediatrics, University Hospital Schleswig–Holstein, University of Lübeck, Germany. Barbara Mitschdörfer: Bundesverband “Das frühgeborene Kind”, Frankfurt, Germany. Silke Mader: European Foundation for the Care of Newborn Infants (EFCNI), Munich, Germany. Tanja K. Rausch and Inke R. König: Institute of Medical Biometry and Statistics, University of Lübeck, Germany.
Name and contact information for the trial sponsor {5b}	Prof. Dr. Wolfgang Göpel on behalf of the University Hospital of Schleswig–Holstein (UKSH), Department of Pediatrics Street: Ratzeburger Allee 160, Postal zip code 23538 City: Lübeck Country: Germany
Role of sponsor {5c}	The pro.LISA trial is a sponsor-investigator study. Therefore, the sponsor is responsible for all aspects of the study. The funder (BMBF) is not responsible for trial-related aspects.

## Introduction

### Background and rationale {6a}

Less invasive surfactant administration (LISA) describes intratracheal surfactant administration via a small diameter tube to spontaneously breathing infants [1]. The current guidelines recommend surfactant therapy in preterm infants if the fraction of inspired oxygen (FiO_2_) is higher than 0.30 and LISA as the optimal mode of administration for preterm infants on continuous positive airway pressure (CPAP) [2]. This recommendation is based on a number of randomised controlled trials and meta-analyses [3–7]. The use of prophylactic LISA in infants with FiO_2_ at or below 0.30 was not yet tested in randomised controlled trials.

Observational data from the GNN indicate that in infants with gestational age 25–28 weeks and FiO_2_ ≤ 0.30, LISA treatment rates are as high as 50%. Reasons for this high rate of LISA despite the lack of data and high costs of surfactant therapy are unclear.

Prophylactic surfactant therapy via an endotracheal tube is not superior to early CPAP and selective surfactant treatment of infants requiring intubation [8]. Furthermore, even LISA causes some stress for preterm infants, since the trachea is intubated with a small diameter tube, which can induce discomfort, coughing, desaturations, tracheal obstruction, and bradycardia. Possible benefits of LISA prophylaxis include a lower rate of intubation and mechanical ventilation and better long-term lung function [9]. The existing uncertainties concerning LISA prophylaxis prompted us to design and initiate the pro.LISA trial.

### Objectives {7}

The primary hypothesis of the pro.LISA study is that there is a difference in forced expiratory volume within 1 s (FEV_1_) at the age of 5 years between the intervention group (infants receiving prophylactic surfactant via LISA) and the control group (no prophylactic surfactant via LISA). Secondary hypotheses are that there are differences with respect to other lung function parameters, endurance, motor development, intelligence, and susceptibility to infectious lung disease.

### Trial design {8}

The pro.LISA study is a prospective, randomised, controlled, parallel-group, single-blinded, multicentre, national trial. Patients will be assigned in a 50:50% ratio to the intervention and control groups. The purpose of the trial is to show the superiority of the intervention with regard to the primary endpoint.

## Methods: participants, interventions, and outcomes

### Study setting {9}

Neonatal intensive care units in Germany are enrolled as participating sites. A list of participating centres is given in the “Acknowledgements” section.

### Eligibility criteria {10}

The following are the inclusion criteria:

- Preterm infants with gestational age between 25 weeks and 0 days and 28 weeks and 6 days
- Age < 60 min
- Spontaneous breathing
- Pulse oximetric measured oxygen saturation ≥ 90% at FiO_2_ ≤ 0.30
- Written informed consent of at least one legal guardian

The following is the exclusion criteria:

- Lethal malformations

### Who will take informed consent? {26a}

Pregnant women who are admitted to a centre participating in the pro.LISA trial with threatening preterm delivery before 29 weeks of gestation will be approached. Both parents/legal guardians will be asked for consent before enrolment. In case of an emergency situation (e.g. emergency C-section), the consent of only one parent is sufficient for enrolment. The other parent will be approached as soon as possible. Informed consent will be taken by qualified staff members of the participating site.

### Additional consent provisions for collection and use of participant data and biological specimens {26b}

Parents/legal guardians give consent for the collection and analysis of data to study the safety and efficacy of prophylactic LISA treatment. This includes the use of data for regulatory purposes and ancillary analyses (e.g. meta-analyses). All sites of the pro.LISA trial are part of the German Neonatal Network (GNN). In the GNN, deoxyribonucleic acid samples of participating children are collected. Parents are asked for separate consent for enrolment in the GNN.

### Interventions

#### Explanation for the choice of comparators {6b}

For preterm infants breathing spontaneously on CPAP, surfactant therapy if FiO_2_ exceeds 0.30 is recommended by the current guidelines [2] and was chosen as the control group procedure. In the intervention group, preterm infants will be treated with prophylactic LISA surfactant regardless of FiO_2_ in the first hour of life. We expect that approximately 50% of control group infants will develop FiO_2_ exceeding 0.30 within the first 3 days of life and will therefore be treated with LISA. If similar rates of additional LISA administrations within the first 3 days of life will be necessary in the intervention group will be analysed in the pro.LISA trial.

#### Intervention description {11a}

##### Intervention group

A small diameter tube (according to local standards) is placed via laryngoscopy in the trachea of the infant. Surfactant (100–200 mg/kg body weight) is administered while the baby is breathing spontaneously. Thereafter, the small diameter tube is removed and CPAP with positive end-expiratory pressure level of at least 6 cm H_2_O is continued. The intervention takes about 1 to 3 min.

##### Control group

In the control group, CPAP with a positive end-expiratory pressure level of at least 6 cm H_2_O is continued. LISA is only given if FiO_2_ increases to more than 30% or if the responsible physician observes respiratory distress of the infant.

#### Criteria for discontinuing or modifying allocated interventions {11b}

The intervention (prophylactic surfactant) will be applied immediately after enrolment. No criteria for modifications were defined.

#### Strategies to improve adherence to interventions {11c}

Interventions are documented in the intervention and control groups immediately after randomisation. Due to the short timeframe for the intervention, and the intensive care setting, no strategies to improve adherence were defined.

#### Relevant concomitant care permitted or prohibited during the trial {11d}

The only prohibited therapy is prophylactic surfactant in the control group.

#### Provisions for post-trial care {30}

Patients who are enrolled in the study are covered by patient insurance (Newline Group).

### Outcomes {12}

The primary endpoint of this study is FEV_1_ at the age of 5 years determined by spirometry and adjusted for age, sex, height, and ethnicity according to Global Lung Function Initiative standards (FEV_1_ *z*-scores) [10]. Analysis of the primary endpoint will be done by comparing the intervention and control groups of the full analysis set. Secondary efficacy endpoints at the age of 5 years are as follows:

- FVC *z*-score according to [10]
- FEV_1_/FVC *z*-score according to [10]
- Length in metres of running track in 3-min running test according to [11]
- Cerebral palsy defined as Gross Motor Function Classification System value > 1
- Score in Movement Assessment Battery for Children
- Intelligence assessed by Wechsler Preschool and Primary Scale of Intelligence (WPPSI) IV
- Obstructive bronchitis (treated with inhaled or other drugs) in the last 12 months

Spirometry measures (FEV_1_, FVC, and FEV_1_/FVC-scores) and the length of the running track will be aggregated as mean values. Movement Assessment Battery for Children and WPPSI-IV-scores will be aggregated as median scores. Cerebral palsy and obstructive bronchitis will be analysed as proportions. Safety endpoints will be assessed at discharge from the hospital and at the age of 5 years and include death and complications of premature birth. All adverse events and serious adverse events will be reported.

The following safety endpoints will be assessed at discharge from the hospital:

1. Death
2. Intraventricular haemorrhage (IVH, each grade)
3. IVH grade III or IV
4. Periventricular leukomalacia (PVL)
5. Abdominal operation due to focal intestinal perforation (FIP)
6. Abdominal operation due to necrotising enterocolitis (NEC)
7. Laser surgery, cryocoagulation, or anti-VEGF treatment of retinopathy of prematurity (ROP)
8. Operation due to a persistent ductus arteriosus (PDA)
9. Pneumothorax
10. Bronchopulmonary dysplasia (BPD, need of O_2_ or breathing assistance with 36 weeks p.m.)
11. Death or BPD
12. Duration of mechanical ventilation (in days)
13. Duration for need of O_2_ (in days)
14. Duration of need of any breathing assistance (ventilation or CPAP) (in days)
15. Mechanical ventilation during stay in hospital
16. Mechanical ventilation during the first 72 h of life
17. Herniotomy
18. Abdominal operation due to other reasons
19. Lung bleeding where transfusion or intubation is required
20. Body weight
21. Body length
22. One of the endpoints in 1–10.

The following safety endpoints will be assessed at the age of 5 years:

1. General health
2. Development compared to children of the same age
3. Systolic blood pressure (1st measurement)
4. Systolic blood pressure (2nd measurement)
5. Medium blood pressure (1st measurement)
6. Medium blood pressure (2nd measurement)
7. Diastolic blood pressure (1st measurement)
8. Diastolic blood pressure (2nd measurement)
9. Heart rate
10. Breathing rate
11. SpO_2_
12. Systolic blood pressure after running test
13. Diastolic blood pressure after running test
14. Medium arterial pressure (MAD) after running test
15. Heart rate after running test
16. Breathing rate after running test
17. SpO_2_ after running test

Safety endpoints will be compared in the safety analysis dataset. The method of aggregation for safety endpoints until discharge will be proportions for numbers 1–11, 15–19, and 22 and median values for 12–14 and 20–21. The method for aggregation of data at 5-year follow-up will be proportions for items 1–2 and mean values for all other endpoints. All statistical analyses are described in detail in the statistical analysis plan, which will be finalised before the randomisation of the last patient in the study.

### Participant timeline {13}

**Table:** 

	In hospital					After discharge (years)				
	Before birth until 60 min	< 60 min	72–84 h	36 weeks p.m	Discharge	1	2	3	4	5
Eligibility screen	x									
Informed consent	x									
Allocation		x								
Intervention group: prophylactic surfactant immediately after allocation		x								
Control group: no prophylactic surfactant after allocation		x								
Baseline assessment	x									
Outcome assessment (safety)			x	x	x	x	x	x	x	x
Adverse events		x	x		x					
Outcome assessment (efficacy)										x

### Sample size {14}

The clinical aim is to detect a mean difference in FEV_1_ *z*-scores of 0.3 at the age of 5 years [9]. Based on the values of the GNN study, the *z*-score standard deviation is expected to be 1.07. The significance level alpha is set to 0.05 (two-sided) and power to 0.8. To detect this effect using a two-sample *t*-test, it requires data from 201 patients per group. The total sample size is calculated on the following assumptions: adjustment for multiple births (10%), rate of patients dying before discharge (2%), loss to follow-up rate (28%), and the rate of patients who will not be able to accomplish lung function tests at the age of 5 years (10%). Based on these estimates, we calculated that data from 349 patients per group are needed (698 patients in total, nQuery 7.0).

### Recruitment {15}

At the time of this report, patients are recruited in 20 neonatal intensive care units in Germany. Names of site investigators and affiliations are given in the “Acknowledgements” section. Enrolment of one or two patients per month per centre will ensure enrolment of the target sample size within 30 months. However, the pro.LISA study group will recruit and initiate additional sites.

### Assignment of interventions: allocation

#### Sequence generation {16a}

A stratified permuted block randomisation is used with stratification by gestational age (25 and 26 weeks vs. 27 and 28 weeks), multiple birth status, and participating study centres. The randomisation lists are prepared by the Institute of Medical Biometry and Statistics.

#### Concealment mechanisms {16b}

Sequentially numbered opaque, sealed envelopes are provided to participating centres for the allocation of patients.

#### Implementation {16c}

Generation of allocation sequence and provision of randomisation envelopes is done by persons and institutions who are not involved in patient enrolment.

### Assignment of interventions: blinding

#### Who will be blinded {17a}

Blinding of physicians and nurses for the intervention is not possible in this study. Parents and outcome assessors of the primary outcome at age 5 years will be blinded to the procedure.

#### Procedure for unblinding if needed {17b}

Since physicians are not blinded, this will be not relevant.

### Data collection and management

#### Plans for assessment and collection of outcomes {18a}

Data of the pro.LISA trial are collected in an online database in the Castor EDC system [12]. Assessment forms for follow-up until 5 years of age are defined and implemented in the database. The time of regular assessments is given in the “Participant timeline {13}” section, and methods for outcome measures are given in the “Outcomes {12}” section.

#### Plans to promote participant retention and complete follow-up {18b}

We expect that follow-up until discharge from the hospital will be very close to 100%. For long-term follow-up, we are planning to contact parents every 6–12 months via phone or mail to minimise loss to follow-up.

#### Data management {19}

All study data are collected at a central online database. Details for data security and storage are given on the website [12]. To improve data quality at data entry, we established range checks for data values in the pro.LISA online database.

#### Confidentiality {27}

Since we need the contact information of parents for follow-up, these data are entered in the pro.LISA online database. Access to the pro.LISA online database is limited to investigators (who have only access to patient data of their own site) and qualified personnel from the study centre (University of Lübeck).

#### Plans for collection, laboratory evaluation, and storage of biological specimens for genetic or molecular analysis in this trial/future use {33}

In the pro.LISA trial, no biological specimens are collected. We encourage parents to participate in the GNN cohort study in addition to the pro.LISA trial. In the GNN cohort study, deoxyribonucleic acid samples of participating infants are collected.

## Statistical methods

### Statistical methods for primary and secondary outcomes {20a}

All details of statistical analyses will be specified in the statistical analysis plan, which will be finalised before the inclusion of the last patient. The primary and other efficacy endpoints will be analysed in the full analysis population. Patients who died before the 5-year follow-up and patients who were not able to complete the lung function test will be excluded from the full analysis set. The primary endpoint will be tested in a generalised estimating equation model assuming Gaussian error and exchangeable correlation matrix using a two-sided Wald test at a significance level alpha of 0.05.

#### Analysis population

**Table:** 

	Randomisation	SA	ITT	FA	PP
Violation of major exclusion criteria	IV	IV	IV	IV	–
Violation of major exclusion criteria	CO	CO	CO	CO	–
Wrong treatment	IV	CO	IV	IV	–
Wrong treatment	CO	IV	CO	CO	–
Other major protocol deviation	IV	IV	IV	IV	–
Other major protocol deviation	CO	CO	CO	CO	–
No data for FU	IV	IV	IV	–	–
No data for FU	CO	CO	CO	–	–
None of above	IV	IV	IV	IV	IV
None of above	CO	CO	CO	CO	CO

*SA* Safety analysis set, *ITT* Intention-to-treat population, *FA* Full analysis set, *PP* Per-protocol population, *IV and CO* Randomised or analysed in the intervention and control group, respectively, “–” excluded from the analysis

Safety endpoints will be evaluated in the safety analysis (SA) set. The primary endpoint and other efficacy endpoints will be evaluated in the full analysis (FA) sets. Where possible, secondary endpoints will be analysed in the intention to treat (ITT) analysis population.

As a sensitivity analysis, all efficacy endpoints will additionally be evaluated in other analysis populations including a per protocol population.

### Interim analyses {21b}

No interim analyses are planned.

### Methods for additional analyses (e.g. subgroup analyses) {20b}

A sensitivity analysis will be done for the primary endpoint adjusted for smoking during pregnancy and any breastfeeding during the stay in the hospital. No further subgroup analyses are planned.

### Methods in analysis to handle protocol non-adherence and any statistical methods to handle missing data {20c}

The primary and secondary endpoints can only be examined if the patients are able to conduct the tests. Patients who are not able to conduct the tests and patients who died before the assessment will be excluded from the full analysis set.

Other scenarios for not conducting a follow-up are the following: unattainability of a patient, absence at the follow-up appointment, or the parents do not want to attend the follow-up examination. For these scenarios, missing values might not be related to the outcome and be comparable to the observed values in the intervention and control groups, and these scenarios assume that values are missing at random or missing completely at random. Thus, a multiple imputation for the endpoints will be done.

No other imputations of missing data will be performed, and all other analyses will be based on complete cases.

### Plans to give access to the full protocol, participant-level data, and statistical code {31c}

After analysis and publication of the primary and secondary outcome data, full access to de-identified patient-level outcome data, the full protocol of the trial, and the statistical code will be available upon reasonable request.

### Oversight and monitoring

#### Composition of the coordinating centre and trial steering committee {5d}

The trial management group includes the principal investigator, clinical and administrative research associates, research nurses, data managers, and statisticians. The trial management group has weekly meetings. Reports concerning site-specific enrolment numbers are provided to participating sites and the data and safety monitoring board every month. Personnel meetings of the whole study group are scheduled every 6 months.

#### Composition of the data monitoring committee, its role, and reporting structure {21a}

The data and safety monitoring board include an independent statistician, a clinical expert in neonatology, and two representatives of preterm infant parent organisations. The role of the data and safety monitoring board is to monitor the progress of the trial and to ensure that the conduct of the trial is safe and ethically acceptable. The data and safety monitoring board reports to the sponsor/investigator who has full responsibility regarding any decision concerning the continuation or stopping of the trial.

#### Adverse event reporting and harms {22}

Preterm infants are a vulnerable group of patients and a high number of adverse events was anticipated in the pro.LISA trial. Typical complications of preterm birth (e.g. intraventricular haemorrhage, surgery due to necrotising enterocolitis) were defined as adverse events, are collected systematically, and are entered in the pro.LISA online database. Serious adverse events (deaths in particular) are reported to the principal investigator within 24 h either by fax or in the pro.LISA online database. Unexpected harms will be also collected as adverse events or serious adverse events in the online database. Harms will not be classified according to standardised classifications like the “common terminology criteria for adverse events”. Suspected unexpected serious adverse reactions are not expected since more than a million preterm infants have already been treated with surfactant. Suspected unexpected serious adverse reactions will be reported to the principal investigator within 24 h.

Descriptive analyses of adverse events and serious adverse events including IVH, PVL, surgery for PDA, surgery for NEC, surgery for FIP, surgery for ROP, and pneumothorax are provided every month to the data and safety monitoring board and participating sites.

#### Frequency and plans for auditing trial conduct {23}

Trial institutions, facilities, and all data on electronic case record forms must always be available for inspection by an authority. Online monitoring of electronic case record forms is done by the trial management group on a continuous basis. Close out of centres after completed enrolment will be done by personnel visits.

#### Plans for communicating important protocol amendments to relevant parties (e.g. trial participants, ethical committees) {25}

Changes and additions to the protocol will be submitted to the ethics committees of all participating centres and regulators for review and approval. Thereafter, changes are communicated to participating sites and—if necessary—to parents of trial participants.

#### Dissemination plans {31a}

In general, results from the study will only be published after the database has been closed. Exceptions are publications concerning the design of the study and descriptive results of safety analysis after discharge from the hospital of all enrolled infants. The latter is planned, since the primary endpoint will be measured 5 years after discharge of the last enrolled patient. Since short-term complications might be different between the groups, we are planning descriptive outcome analyses of these data after enrolment is completed.

The final report of the trial will be published within a period of 360 days upon completion of the study.

## Discussion

In meta-analyses of randomised controlled trials, surfactant application via LISA is superior to surfactant application via an endotracheal tube in terms of mechanical ventilation within the first 72 h of life, BPD, other complications of preterm birth, and mortality [7]. Furthermore, LISA is now recommended as the preferred mode of surfactant administration in preterm infants with FiO_2_ > 30% [2].

Important uncertainties persist with regard to the possible benefits of LISA in preterm infants with mild respiratory distress syndrome and FiO_2_ ≤ 0.30. Furthermore, studies targeting long-term outcomes of preterm infants who were treated with LISA have not been performed yet.

Prophylactic LISA treatment rates will be 100% in the intervention group of the pro.LISA trial and 0% in the control group. We estimated that within the first 3 days of life, about 50% of control group infants will develop FiO_2_ exceeding 0.30 and will receive LISA. If a similar rate of additional LISA treatment is necessary in the control group will be analysed in the pro.LISA trial. Furthermore, the pro.LISA trial will provide important information if the short-term benefits of prophylactic LISA treatment do exceed the undeniable additional stresses, discomforts, and costs that are induced by a prophylactic LISA procedure. Even if there are no differences with regard to short-term safety data, prophylactic LISA might improve long-term lung function. This endpoint is the target of the primary long-term efficacy endpoint of the pro.LISA trial.

## Trial status

The current protocol version number is Pro.LISA_01_31 version 4 from 11 January 2023. The pro.LISA trial started enrolment in February 2022. The approximate date when recruitment will be completed is the year 2024.

## Data Availability

After analysis and publication of the primary and secondary outcome data, full access to de-identified patient-level outcome data, the full protocol of the trial, and the statistical code will be available upon reasonable request from the principal investigator (WG).

## Abbreviations

BMBF: German Federal Ministry of Education and Research
CPAP: Continuous positive airway pressure
EFCNI: European Foundation for the Care of Newborn Infants
FEV_1_: Forced expiratory volume within 1 s
FiO_2_: Fraction of inspired oxygen
FVC: Forced vital capacity
GNN: German Neonatal Network
LISA: Less invasive surfactant administration
UKSH: University Hospital of Schleswig–Holstein
WPPSI: Wechsler Preschool and Primary Scale of Intelligence
